# Cell attachment on poly(3-hydroxybutyrate)-poly(ethylene glycol) copolymer produced by Azotobacter chroococcum 7B

**DOI:** 10.1186/1471-2091-14-12

**Published:** 2013-05-21

**Authors:** Anton P Bonartsev, Sergey G Yakovlev, Irina I Zharkova, Arasha P Boskhomdzhiev, Dmitrii V Bagrov, Vera L Myshkina, Tatiana K Makhina, Elena P Kharitonova, Olga V Samsonova, Alexey V Feofanov, Vera V Voinova, Anton L Zernov, Yurii M Efremov, Garina A Bonartseva, Konstantin V Shaitan, Michail P Kirpichnikov

**Affiliations:** 1 Faculty of Biology, M.V.Lomonosov Moscow State University, Leninskie gory, 1-12, Moscow, 119236, Russia; 2 A.N.Bach Institute of Biochemistry RAS, Leninskii av., 33-2, Moscow, 119071, Russia; 3 Faculty of Physics, M.V.Lomonosov Moscow State University, Leninskie gory, 1-2, Moscow, 119991, Russia

**Keywords:** Poly(3-hydroxybutyrate), Poly(ethylene glycol), Copolymer, Hydrophilicity, Biocompatibility, COS-1

## Abstract

**Background:**

The improvement of biomedical properties, e.g. biocompatibility, of poly(3-hydroxyalkanoates) (PHAs) by copolymerization is a promising trend in bioengineering. We used strain *Azotobacter chroococcum* 7B, an effective producer of PHAs, for biosynthesis of not only poly(3-hydroxybutyrate) (PHB) and its main copolymer, poly(3-hydroxybutyrate-co-3-hydroxyvalerate) (PHB-HV), but also alternative copolymer, poly(3-hydroxybutyrate)-poly(ethylene glycol) (PHB-PEG).

**Results:**

In biosynthesis we used sucrose as the primary carbon source and valeric acid or poly(ethylene glycol) 300 (PEG 300) as additional carbon sources. The chemical structure of PHB-PEG and PHB-HV was confirmed by ^1^H nuclear-magnetic resonance (^1^H NMR) analysis. The physico-chemical properties (molecular weight, crystallinity, hydrophilicity, surface energy) and surface morphology of films from PHB copolymers were studied. To study copolymers biocompatibility in vitro the protein adsorption and COS-1 fibroblasts growth on biopolymer films by XTT assay were analyzed. Both copolymers had changed physico-chemical properties compared to PHB homopolymer: PHB-HV and PHB-PEG had less crystallinity than PHB; PHB-HV was more hydrophobic than PHB in contrast to PHB-PEG appeared to have greater hydrophilicity than PHB; whereas the morphology of polymer films did not differ significantly. The protein adsorption to PHB-PEG was greater and more uniform than to PHB and PHB-PEG copolymer promoted better growth of COS-1 fibroblasts compared with PHB homopolymer.

**Conclusions:**

Thus, despite low EG-monomers content in bacterial origin PHB-PEG copolymer, this polymer demonstrated significant improvement in biocompatibility in contrast to PHB and PHB-HV copolymers, which may be coupled with increased protein adsorption and hydrophilicity of PEG-containing copolymer.

## Background

The last few decades have been characterized by intensive development of biomedical materials based on biodegradable polymers [1,2]. PHAs are biodegradable and biocompatible polyesters of bacterial origin [3,4]. Unlike most biopolymers, PHAs are produced biotechnologically, which permits the control of chemical structure and physicochemical properties of the produced polymers during biosynthesis. PHB and its copolymers are natural biopolymers that display several unique properties, such as a high biocompatibility with mammalian cells, tissues and organs and the ability to biodegrade without forming toxic byproducts. High levels of PHB accumulation in bacterial cells of biopolymer producers and the solubility of PHB in organic solvents make the process of isolation and deep purification of PHB for biomedical applications relatively simple, while maintaining excellent quality [5-7]. The widest field of PHB and its copolymers application includes surgical implants used in hernioplasty, dentistry, cardiovascular surgery and orthopedic surgery, etc. The biopolymers are used in development of biodegradable sutures, biodegradable screws and staples, periodontal membranes in dentistry, surgical meshes with biopolymer coatings, wound coatings, surgical patches for defects in the intestine, pericardium, or bone tissues and other tissues [6,7]. Unfortunately, the PHB homopolymer has some physicochemical properties that limit its biomedical usefulness. Namely, the solution cast films of PHB have brittle properties, a high crystallinity degree, high hydrophobicity and low rate of biodegradation. These factors limit development, for example, in the formation of artificial blood vessels based on PHB biomaterial [8].

The improvement of polymer biomedical properties, e.g. biocompatibility, by blending or copolymerization is a promising trend in bioengineering [1,2]. The improvement of PHB biocompatibility by copolymerization was developed by various chemical and biotechnological methods. However the chemical methods for biomedical applications suffer from a series of shortcomings: the necessity for deep purification of chemical impurities, limited stereoregularity and synthesis of high-molecular weight polymers as well as limitations in accurate control of physicochemical properties of the produced polymers, toxicity of polymers of non-natural origin and products of their biodegradation. Despite of approximately 100 various hydroxyalkanoic acids that have been detected as components of PHAs and over 300 PHA producers, only a limited number of bacterial PHA producers can synthesize PHB homopolymer or a certain PHB copolymer with a relatively high efficiency (with high biomass and polymer yields), which is available for biomedical-grade polymer production and isolation [3-7]. *Azotobacter chroococcum* is one of the most efficient PHB producers, which able to accumulate up to 80% of dry weight of cells with great biomass growth parameters [7,9]. The effect of carbon nutrition conditions on PHA synthesis was actively studied in the context of the possibility to synthesize not only single-component, but also multicomponent PHAs to improve physicochemical and biomedical properties of biopolymers. For instance, addition of valerate and propionate leads to the production of copolymer PHB-HV with changed physico-mechanical properties [9-12]. Moreover, Shi F. at al. suggested using not only monomeric organic acids or alcohols but also some polymers, e.g. polyethylene glycol (PEG), as additional carbon source for PHB copolymers biosynthesis [13,14]. Polyethylene glycol (PEG), a neutral water-soluble polyether is relatively non-toxic to cellular systems and is absorbed into proteins and the phospholipid head group. PEG is used in processes such as protein modification, cell fusion and organ preservation, etc. [15-17]. In pharmacology and bioengineering, PEG is often used for chemical modification (PEGylation) of polymer nanoparticles, liposomes and biopharmaceuticals. PEGylation simply refers to the decoration of a polymer surface by covalently grafting, blending, or adsorbing PEG chains [18-20]. The chemical PHAs copolymerization or blending with PEG can improve or impair polymer biocompatibility, depending on mechanisms that are also not quite clear yet, whereas the effect of biotechnological copolymerization of PHAs with PEG on polymer biocompatibility is even less clear [21-25].

Here, we produced PEG-containing PHB copolymer by using *Azotobacter chroococcum 7B* and investigated how microbiological PEGylation of PHB affects the physicochemical properties and biocompatibility of produced copolymer.

## Methods

### Materials

Poly(ethylene glycol) 300 g/mol (PEG 300), sodium salt of valeric acid (VA), sodium acetate (SA); components of growth media: K_2_HPO_4_ × 3H_2_O, МgSO_4_ × 7H_2_O, NaCl, Na_2_MoO_4_ × 2H_2_O, CaCO_3_, FeSO_4_ × 7H_2_O, sodium citrate, CaCl_2_, KH_2_PO_4_, sucrose, agar, phosphate-buffer saline (PBS). All materials were purchased from Sigma-Aldrich and used as recommended by the manufacturer.

### Growth conditions

A PHA producer *A. chroococcum* strain 7B, a non-symbiotic nitrogen-fixing bacterium able to overproduce PHB (to 80% of cell dry weight) was used [26-29]. The strain was isolated from the wheat rhizosphere (sod-podzolic soil) and maintained on Ashby’s medium, containing 0.2 g/l K_2_HPO_4_ × 3H_2_O, 0.2 g/l МgSO_4_ × 7H_2_O, 0.2 g/l NaCl, 0.006 g/l Na_2_MoO_4_ × 2H_2_O, 5.0 g/l CaCO_3_, 20 g/l sucrose, and 20 g/l agar. All experiments were performed under laboratory conditions. For PHB synthesis in cells, the culture was grown in shaker flasks (containing 100 ml of the medium) at 30°C in Burk’s medium, containing: 0.4 g/l МgSO_4_ × 7H_2_O, 0.01 g/l FeSO_4_ × 7H_2_O, 0.006 g/l Na_2_MoO_4_ × 2H_2_O, 0.5 g/l sodium citrate, 0.1 g/l CaCl_2_, 1.05 g/l K_2_HPO_4_ × 3H_2_O, 0.2 g/l KH_2_PO_4_, and 20 g/l (50 mM) sucrose as the primary carbon source. For PHB copolymer biosynthesis, the additional carbon sources were added to the culture medium. As a 3-hydroxyvalerate (3HV) precursor in the PHB-HV copolymer chain, VA was added as sodium salts at a concentration of 5 mM after 12 h incubation of the culture. To control M_w_ of PHB and PHB-HV sodium acetate (SA) at a concentration of 6.2 g/l (75 mM) and 4.1 g/l (50 mM), respectively, was added to medium. The concentrations of 5 mM VA and 50–75 mM SA were selected as optimal for production polymer with selected monomer composition and molecular weight according to previous data [9,26]. The reduction PHB and PHB-HV M_w_ by SA adding was essential for production of polymers comparable with low-molecular-weight PHB-PEG. PEG 300 was added initially in the medium at a concentration of 150 mM. It was shown that further increases in PEG concentration inhibited growth, reduced PHA production, molecular weight and 3HV incorporation. Thus, we used the maximal optimal concentration of PEG [14]. The experiment was performed for 72 h. Optical density was controlled by nephelometry. To control strain growth and polymer accumulation in cells a Biomed 1 (Biomed, Russia) light microscope was used. The parameters of the copolymers biosynthesis including biomass yield and polymer yield were determined according to [9,26].

### Production of highly purified biopolymers from bacterial biomass

The polymer isolation and purification from *A. chroococcum* for biocompatibility study comprised the following stages: (1) polymer extraction with chloroform in a shaker for 12 h at 37°C; (2) separation of polymer solutions from cell debris by filtration; (3) polymer precipitation from chloroform solution with isopropanol; (4) subsequent repeated cycles of dissolution in chloroform and precipitation with isopropanol for 4–5 times to remove any additives and contaminants, and (5) drying at 60°C [9,26].

### Molecular weight determination

Molecular weights (M_w_) of PHB, PHB-HV and PHB-PEG were determined by gel permeation chromatography (GPC) using a Waters 1525 pump, connected to four Waters styragel columns (Styragel HT 6E, 4.6 × 300 mm) placed in series. The detection system consisted of a Waters 2414 differential refractive index detector and a UV detector. Chloroform was the eluent, at a flow rate of 1.0 mL/min. Typical sample volumes were 50 μL at a polymer concentration of 2 mg/mL. Narrow polydispersity polystyrene standards (Sigma-Aldrich, USA) were used to generate a universal calibration curve, from which the molecular weights were determined, after correcting for flowrate variations based on the elution volume of the flow-rate marker [30]. The Mw determined by GPC was correlated with data estimated by viscosimetry: the viscosity of the PHB solution in chloroform was measured at 30°C on an RT RheoTec viscometer (RheoTec, Germany); the molecular mass was calculated using the Mark-Kuhn-Houwink equation according to [26].

### Proton nuclear magnetic resonance (^1^H NMR) spectroscopy

**Figure 1:**
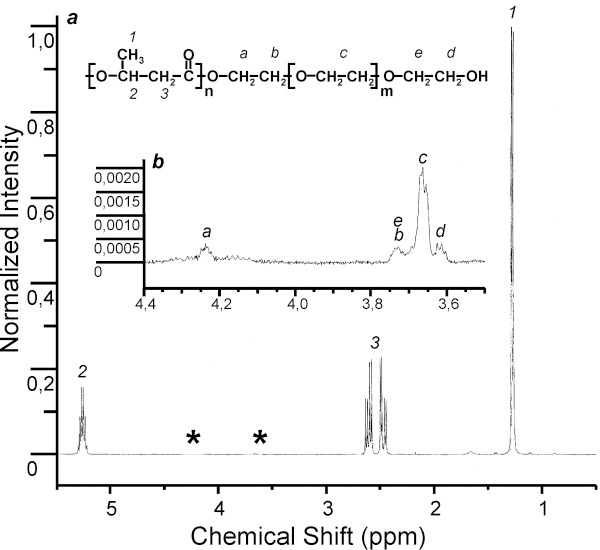
^**1**^**H-NMR spectra of the PHB-PEG. **^1^H-NMR spectra of the PHB-PEG (1): (**a**) PHB chain: 1 is CH_3_(s), 2 is CH(b), 3 is CH_2_(b), s is a side chain, and b is a polymer backbone; * see zoomed graph section on (b); (**b**) PEG chain: a is linking –O–CH_2_ (4.24 ppm), b is following CH_2_ (3.73), c is the integral signal from median [–O–CH_2_–CH_2_–] group (3.66 ppm), e and d are tail –CH_2_– (3.70 ppm) and –CH_2_–OH (3.61 ppm) groups, respectively.

Proton (^1^H) NMR spectra of PHB and its copolymers solutions in deuterated chloroform were recorded in an MSL-300 (Bruker, Germany) spectrometer at a working frequency of 400 MHz. Chemical shifts in parts per million (ppm) were measured from 0.00 ppm relative to the signal of chloroform-d (CDCl_3_) residual protons, 7.27 ppm. The experimental parameters were as follows: 1% (w/v) polymer in chloroform-d, 313 K, 2.5 s acquisition time, and 4000 Hz spectral width. The percent content of elementary 3HV elements in the PHB-HV copolymer was calculated according to the ratio of the integral signal intensity from the 3HV methyl group (0.89 ppm) to the sum of integral signal intensities from the methyl groups of 3HV and hydroxybutyrate (1.27 ppm) (see Figure 1). The percent content of elementary EG elements in the PHB–PEG copolymer was calculated according to the ratio of the sum of integral signal intensities from EG –CH_2_– groups (3.61, 3.70, 3.66, 3.73, 4.24 ppm) to the sum of integral signal intensities from the methyl group of hydroxybutyrate (1.27 ppm) (see Figure 1).

### Polymer films preparation

PHB, PHB-HV and PHB-PEG films were prepared by casting a 3 wt. % chloroform solution of the polymers onto a glass Petri dish. After slow evaporation of chloroform, the remaining solvent in the films was removed by drying the films under vacuum at 50°C for two days. The thickness of polymer films was 50 ± 5 μm. As it was shown earlier the roughness of polymer film surfaces exposed to air is much greater roughness exposed to glass. Such differences are related to conditions of solvent (chloroform) desorption and evaporation from the forming PHB films [31]. Here and below, the surface of the polymer film exposed to Petri dish glass is called the “smooth”; and the surface exposed to air is called the “rough”.

### Differential scanning calorimetry (DSC)

The PHB, PHB-HV and PHB-PEG thermal properties were measured by means of differential scanning calorimetry using a DSC 204 F1 Phoenix (Netzsch, Germany) equipment. About 1–4 mg of polymer film was sealed in a 25 μL aluminium crucible. The samples were heated from 25 to 200°C at a heating rate of 10°C/min in nitrogen atmosphere. Netzsch calibration set (KNO_3_, In, Bi, Sn, Zn, CsCl, Hg, C_6_H_12_ high purity samples) was used for precise temperature and enthalpy calibrations in temperature range -100°C – 600°C according to the manufacturer instructions [12,32,33]. The onset and peak temperature of the change in heat capacity was designated as the $T_{m^{\text{onset}}}$ and $T_{m^{\mathrm{peak}}}$ melting points. The crystallinity of PHB component (X_c_) can be calculated by the following [34]

$$X_{c}=\frac{\left(\Delta H_{m}+\Delta H_{r}\right)}{\Delta H^{0}{}_{m\left(\mathrm{PHB}\right)}}\times 100\%,$$

where Δ*H*_*r*_, Δ*H*_*m*._ are the enthalpy contributions caused by recrystallization and melting of investigated sample, respectively, Δ*H*^*0*^_*m(PHB)*_ is the theoretical value for the thermodynamic melting enthalpy, which would be obtained for a 100%-crystalline PHB sample (146.6 J/g) [35]. All calculations were performed for the first heating cycle [12,30,32,34].

### Contact angle tests

The hydrophilicity of polymer surface was evaluated by measuring the water contact angle formed between water drops and the surface of the samples using a Contact Angle Meter 110 VAC (Cole-Parmer, USA). For this purpose, a drop of 10 μl of milliQ water was mounted on the surface with a microsyringe and quickly measured by the Contact Angle Meter. The advancing contact angle was measured for 8 drops on both sides at a temperature of 25°C, and the average was calculated from the data. The apparent contact angle can be measured exactly (accuracy of the method is 0.1%), but as a result of the roughness of the sample a typical statistical error was in the range of 1-2% [35].

### Water uptake test

Films were cut into 10 × 10 mm samples and immersed in deionized water at 37°C. At predetermined time intervals, hydrated samples were picked up and weighed after the surface water was blotted away with Kimwipes. The water contents were then calculated on the basis of the weight difference of the film before and after swelling. The percentage of water uptake was calculated using the following equation

$$\mathrm{WU}\%=\frac{\left(W_{w}-W_{d}\right)}{W_{d}}\times 100\%$$

where WU% is water uptake (%), W_d_ and W_w_ are the weights of the sample film before and after being immersed in water, respectively [25].

### Atomic force microscopy

Microphotographs of the surface of PHA films were obtained be means of atomic force microscopy (AFM). The AFM imaging was performed with Solver PRO-M (Zelenograd, Russia). For AFM imaging a piece of the PHB film (~2 × 2 mm^2^) was fixed onto a sample holder by double-sided adhesive tape. Silicon cantilevers NSG11 (NT-MDT, Russia) with a typical spring constant of 5.1 N/m were used. The images were recorded in semi-contact mode, a scanning frequency of 1–3 Hz, scanning areas from 3 × 3 to 20 × 20 μm^2^, and topography and phase signals were captured during each scan. The images were captured with 512 × 512 pixels. Image processing was carried out using Image Analysis (NT-MDT, Russia) and FemtoScan Online (Advanced technologies center) software.

The average roughness, R_a_, was calculated to describe film surfaces

$$R_{a}=\frac{1}{N}\sum_{n=1}^{N}\left|r_{n}\right|$$

This parameter was calculated by three scan areas of 20 × 20 μm^2^ (512 × 512 points). Additionally, several scans at higher resolutions (e.g., 5 × 5 μm^2^ (512 × 512 points)) were obtained for each sample for more detailed description of the polymer surface [31].

### Protein adsorption

The polymer films were incubated in Dulbecco’s Modified Eagle Medium (DMEM) containing 10% (v/v) fetal bovine serum (Invitrogen, USA), at 37°C for 24 h. After incubation, the samples were incubated in a buffer constituted by TRIS 10 mM, EDTA 1 mM and SDS 0.1% (v/w); the samples were mixed for 6 h at 3–4°C. This procedure permits to remove all proteins. In this way, the proteins adsorbed on the surface were removed from the sample and were determined by protein assay using Bradford Reagent (Sigma-Aldrich, USA) with a spectrophotometer Ultrospec 1100 pro (Amersham Biosciences Corp., USA). The experimental data are presented as the amount of protein adsorbed per unit surface area (cm2) of polymeric membranes [34]. To visualize adsorbed protein on polymer film surface FITC-BSA (Sigma-Aldrich, USA) was used. Protein adsorptions by intensively washed (using MilliQ water) and dried polymer films were investigated by incubating the films in a solution of FITC-BSA in 10 mM TRIS for 2 h at 37°C. The analyses of adsorbed protein on the “smooth” surface of polymer films were carried out by fluorescence microscopy using Axiovert 200 M fluorescent microscope with a digital AxioCam camera running the Zeiss LSM Image Browser 4.2.0 software (Carl Zeiss MicroImaging GmbH, Germany).

### Cell culture

The simian fibroblasts of COS-1 cell line (Biolot, Russia) was used for polymer biocompatibility testing [36,37]. The cells were cultivated in DMEM (Dubecco’s Modified Eagle Medium, Invitrogen, USA) with high glucose content (4.5 g/l) supplemented with 10% fetal calf serum (FCS), 100 IU/ml penicillin, and 100 μg/ml streptomycin solutions (Invitrogen, USA). Cells were incubated at 37°C in a humidified 5% CO_2_ atmosphere and the medium was changed every day. Fibroblasts were released before confluence with trypsin-versen solution (0.05% (v/w) trypsin and 0.02% (v/w) EDTA in PBS) (Serva, Germany) and counted with Coulter Counter Z1 (Beckman Coulter, USA).

### Cell viability test

To analyze polymer biocompatibility cell attachment and proliferation on PHB, PHB-HV and PHB-PEG polymer films were studied. The initial cell attachment to material surface and subsequent cell proliferation on (in) the material show quantitative integral indirect data on material properties that can be used for evaluation of living cells compatibility with the examined material: the biochemical reactivity of the material, the release of toxic products from the material, the availability of surface morphology of the material for cell growth, the biophysical surface properties (e.g. charge, hydrophilicity) of the material etc. Therefore, the cell viability tests for in vitro cell attachment and proliferation on the various materials are widely used to analyze biocompatibility of these materials [1,2,6,7,20,23,25,34],[36,37]. Eight samples for each polymer were placed in 96-well tissue culture plates and a cell suspension of 5000 cells/ml was directly seeded on every sample. Polymer films were placed in the wells with the “rough” surface upwards. The same amount of cells was plated in six empty polystyrene wells for each plate as a negative control. Plates was incubated for 24, 48, 72 and 96 h. Cell proliferation and viability were measured by the cell proliferation reagent based on the cleavage of the tetrazolium salt to soluble formazan salt by mitochondrial activity of viable cells (XTT Cell Proliferation Kit, Biological Industries, Israel). At the end of the experimental time, polymer films with attached cells were gently and quickly transferred from wells of incubated tissue culture plate to respective wells of new plate with preliminarily added 100 μl fresh medium. Then 50 μl XTT reagent solution was added to the cell monolayers on polymer films in each well, and the multi-well plates were incubated at 37°C for a further 4 h. Polymer films were removed and samples were quantified spectrophotometrically at 450 nm with reference wavelength at 640 nm. Viable cell numbers on films were then determined from the standard curve based on their XTT absorbency. Results were reported as optical density (OD).

### Statistical analysis

Statistical evaluation of data was performed using the software package SPSS/PC + Statistics™ 12.1 (SPSS). After verifying that the data were normally distributed and showed a homogeneity of variance, the non-parametric Kruskal–Wallis test was used to highlight any significant difference for in vitro and in vivo results between tested polymers for each experimental time by applying the following comparisons: *PHB, PHB-HV and PHB-PEG versus PLA, ^#^PHB-HV and PHB-PEG versus PHB. The Mann–Whitney *U*-test was used to compare results between experimental times for each tested polymer. Data were reported as the median ± SD at a significance level of P <0.05.

## Results and discussion

### PHB-HV and PHB-PEG copolymers production

Data on PHB and its copolymers biosynthesis by the A. chroococcum 7B culture grown in a medium containing sucrose as the primary carbon source and supplemented with valeric acid and PEG 300 as additional sources of carbon for synthesis of the copolymers and sodium acetate for M_w_ control are listed in Table 1. The data indicates that use of additional carbon sources, as well as valeric acid and PEG 300, leaded to PHB copolymers production.

**Table 1:** Growth conditions and characteristics of produced biopolymers: PHB, PHB-HV, and PHB-PEG

**Polymers**	**Growth conditions and characteristics**			**Composition in polymer**			**Molecular weight**	
	**Substrates (concentration, mM)**	**Biomass yields (g/l ± SD)**	**Total PHA content, (wt.% ± SD)**	**3HV content, mol%**	**PEG content, mol%**	**Molar ratio PEG/PHB**	**Molecular weight (M**_**w**_**, ×10**^**3**^**)**	**M**_**w**_**/M**_**n**_
PHB	Sucrose (50 mM) + sodium citrate (75 mM)	4.2 ± 0.4	69.3 ± 4.2	0	0	0	178	1.85
PHB-HV	Sucrose (50 mM) + VA (5 mM) + sodium citrate (50 mM)	3.7 ± 0.3	68.1 ± 3.5	5.7	0	0	172	2.04
PHB-PEG	Sucrose (50 mM) + PEG 300 (150 mM)	2.2 ± 0.4	34.2 ± 2.7	0	0.33	0.96	160	1.91

When sucrose is used as the sole carbon source for biopolymer synthesis, the PHA formed by A. chroococcum was a high molecular weight PHB (up to 1600 kDa) [9,26,27]. Earlier, we have shown that 3HV is incorporated into the PHB–HV copolymer when using valeric and propanoic acids as additional carbon sources [9]. It has been shown that the co-substrate, e.g. alkanoic acids, is the main factor in determining the PHA composition. As a rule, organic acids or alcohols with an odd number of carbon atoms are used as either primary or additional sources of carbon to produce copolymers by microbiological synthesis [3,4]. The maximal 3HV content in the copolymer (21.6 mol %) was obtained when using 20 mM sodium salt of VA [9]. It was shown for bacteria of the genus *Azotobacter* that valeric acid is incorporated into the copolymer via the β-oxidation pathway: VA → valeryl-CoA → 3-ketovaleryl-CoA → D-3-hydroxyvaleryl-CoA → 3HV. In this case, sucrose was used as the main carbon source. The molecular weight of the synthesized PHB–HV was lower than that of PHB homopolymer; presumably, this is connected to the addition of organic acids to the sugar-containing growth medium [38]. Early we confirmed HV incorporation into copolymer chains via ^1^H NMR. The ^1^H NMR spectrum of PHB-HV displays the signal of the 3HV methyl group at a chemical shift of 0.89 ppm versus the spectrum of the PHB homopolymer, which lacked this signal. The analysis of ^1^H NMR spectra indicated that the copolymer is a multi-block copolymer, because the signal power of a proton of an esterified β-carbon group is directly proportional to signals of the 3HV methyl group at 0.89 ppm and the 3-HB methyl group at 1.27 ppm [9]. Addition of VA to medium caused relatively moderate decrease in M_w_ of produced copolymer that can be explained by some inhibitory action of carboxylic acids to polymer biosynthesis [9]. But addition of SA leaded to further decrease of M_w_, which is required for polymers comparison (see Methods). Thus, the addition of valeric acid to cultivation medium makes it possible to produce the PHB-HV copolymer with various 3HV contents in the polymer chain.

Addition of PEG-300 (150 mM) to the growth medium also generated EG monomer incorporation into the PHA polymer. Incorporation of EG elements into the PHA polymer was confirmed by ^1^H NMR spectroscopy of PHB–PEG copolymer, as shown in Figure 1a,b. Five weak ^1^H NMR signals at 3.66 ppm (the highest signal) and at 3.61, 3.70, 3.73, and 4.24 were observed that correspond to protons of EG repeat units. The signals at 4.24 and 3.73 ppm were assigned to protons a and b, respectively, of esterified PEG chain segments; peaks at 3.61 and 3.70 ppm were due to protons e and d of terminal free hydroxyl EG units. As seen in Figure 1b, the highest peak was the sum of signals from protons of median EG units of PEG. PHB and PHB-HV formed in the absence of PEG did not show 1H NMR signals in the 3.6-3.8 ppm spectral region [9]. The above results are consistent with the formation of PHA chains that are covalently linked at the carboxylate chain terminus to PEG chain segments. Thus, obtained copolymer is di-block copolymer of PHB and PEG and PEG is attached only to one end of the PHB chain. The maximum incorporation of EG monomers (0.33 mol%) in PHA was obtained with PEG-300 at 150 mM concentration. This value indicates that there are 0.96 molecules of PEG-300 per 1 molecule of PHB (M_w_ = 1.6 × 10^5^). We have introduced this dimensionless parameter, the PEG/PHB molar ratio, as the number of PEG molecules divided by the number of PHA molecules. This parameter facilitates a better understanding of our data.

We observed that simultaneous addition of PEG-300 and sucrose at the initial time point led to a higher PEG content in produced copolymers in contrast to adding PEG-300 after 18 hours, at which point the PHB polymer chain was partially synthesized (data not shown). Adding PEG to the medium also resulted in a significant drop in the molecular weight of produced polymers, as shown in Table 1.

In light of the above, the addition of PEG into culture medium causes a change in PHB biosynthesis involving the enzyme system and results in the formation of a PHB–PEG di-block copolymer where the carboxylate (-COOH) terminus of PHB chains are covalently linked by an ester bond to a PEG chain. PEG attachment to the PHB chain probably occurs during the synthesis of PHA polymers, suggesting possible interaction of PEG with PHB synthase enzyme and the polymer itself. The miscible nature of PEG with PHB and production of PHB/PEG blends was reported earlier [39]. Previously it was shown that this reduction of PHB molecular weight could be attributed to PEG limiting the polymer chain length [14,40]. PEG chain attachment with a covalent bond (resonance at 4.24 ppm) at the terminal position of a PHB chain could lead to break in the elongating PHA chain. The formation of low molecular weight PHB-PEG copolymer by A. chroococcum may be attributed to the interaction of PEG with the PHA molecules itself, as was the case for A. eutropha [13] and P. oleovorans [41]. Earlier, chemical synthesis of PHAs-PEG copolymers has also been reported. PHB-PEG and poly(3-hydroxybutirate-co-4-hydroxybutyrate)-PEG copolymers were produced and their physicochemical properties were examined. However, the molecular weight (Mw) of the copolymers was much lower than natural PHAs, which limits biomedical application of synthetic PHAs-PEG biopolymers [21,22].

The data indicates also that use of all additional carbon sources caused considerable inhibition of cell growth, decrease of polymer content in cells, and consequently, a decrease in polymer yield. Earlier, we have shown that by adding various carboxylic acids (propanoic, hexanoic, heptanoic etc.,) and sodium acetate to the culture medium also resulted in the inhibition of strain growth and polymer accumulation [9,26,27]. Moreover, the level of this decrease depended not only on concentration but also on the nature of the additive. Thus, the biomass yield in medium supplemented with 150 Mm PEG 300 was 2.2 g/l, whereas addition of VA at the same concentration (150 mM) caused a total inhibition of strain growth (data not shown).

### Physico-thermal properties of copolymers

**Figure 2:**
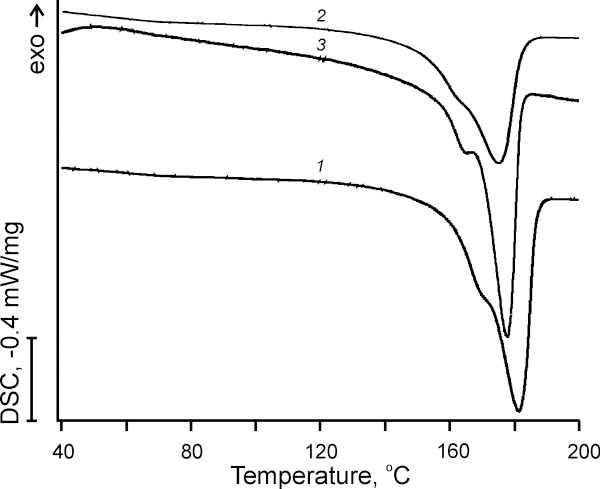
**DSC thermograms of produced biopolymers.** DSC thermograms of produced biopolymers: (1) PHB homopolymer; (2) PHB-HV copolymer; and (3) PHB-PEG copolymer.

Introduction of 3HV into the PHA polymer chain caused significant changes in the physico-chemical characteristics of our produced copolymers. We observed a decrease in crystallinity degree and melting temperature, as shown in Figure 2 and Table 2, which are in agreement with data in the literature [11]. It was shown that among other physico-chemical parameters a crystallinity of PHB copolymers played the most important role in polymers in vitro biocompatibility [34]. DSC curves of Figure 2 revealed the thermal behavior of the polymers: PHB, PHB-HV and PHB-PEG. All polymers were characterized by a melting peak typical of semi-crystalline polymers, as shown in Figure 2. Analysis of DSC curves showed that 3HV incorporation in PHB chain caused: a) a great decrease in area of the PHA melting peak indicating a decrease in total crystallinity degree; b) a shift of the PHA melting peak to an area of lower temperature indicating a decrease in melting temperature; c) a less marked splitting of the main peak in the melting endoterms indicated a more ordered packing of polymer chains in copolymer in comparison with homopolymer, as shown in Figure 2 and Table 2. The PEG incorporation into PHB caused: a) a decrease in total crystallinity degree (on 17% for PHB-PEG in comparison with PHB); b) a slight decrease in melting temperature (on 3.6°С for PHB-PEG in comparison with PHB), as shown in Figure 2 and Table 2. Thus, despite the low incorporation level of PEG into PHB chain, the crystallinity and the melting temperature of produced PHB-PEG copolymer changed markedly that indicates a significant effect of PEG linking to the PHB chain terminus on polymer physico-thermal properties and internal structure of polymer matrix.

**Table 2:** Physico-thermal properties of produced biopolymers: PHB, PHB-HV, and PHB-PEG

**Polymers**	**Melting temperature, onset and peak (**$T_{m^{\mathrm{onset}}}$**/**$T_{m^{\mathrm{peak}}}$**,°C)**	**Crystallinity (*X***_**c**_**, %)**
PHB	161.0/181.4	68.0
PHB-HV	150.6/175.4	50.0
PHB-PEG	157.4/177.8	59.1

### Water related properties of copolymers

The water contact angles obtained on the PHB, PHB-HV and PHB-PEG films and water consumption of the polymers were summarized in Table 3. The surface free energy components and total surface free energy were calculated from contact angles of water and other liquids (DEG, TEG, PEG 300) and were also shown in Table 3. As seen in Table 3, despite the considerable change in physico-thermal characteristics of the PHB-HV copolymer relative to the PHB homopolymer, water-related properties (e.g., contact angle and water consumption) changed little. The small difference in water related characteristics between PHB-HV and PHB homopolymer could be explained by various mechanisms of water-polymer interaction. On the one hand, increased volume of amorphous regions in PHB-HV can promote water diffusion into the polymer matrix. On the other hand, the relatively high 3HV content in PHB-HV (as well 3-hydroxyhexanoate content in PHB-HHx) copolymer increases the number of methyl groups relative to the increase of carbonyl groups in the polymer; at the same time, relatively high 3HV content decreases the oxygen containing moieties on the polymer surface that tend to be more hydrophobic [11,30,42].

**Table 3:** Contact angles and surface free energy of polymer films

	**Water contact angle (θ, °)**		**Total surface free energy (γ**_**S**_**)**	**Surface free energy, dispertion component (**$\gamma_{S^{d}}$**)**	**Surface free energy, polar component (**$\gamma_{S^{d}}$**)**	**Water uptake (w/w, %)**
**Polymer**	**“Smooth surface”**	**“Rough” surface**	**“Smooth surface”**			
PHB	70.5 ± 3.5	75.3 ± 6.1	41.1	29.8	11.3	0.9
PHB-HV	70.2 ± 2.1	77.7 ± 2.6	45.2	17.7	27.5	2.8
PHB-PEG	52.3 ± 7.5^#^	62.9 ± 6.5^#^	49.8	7.5	42.2	30.5

In contrast to the PHB-HV copolymer, the incorporation of PEG in PHB resulted in significant changes of water related properties, as shown in Table 3. The presence of PEG fragments in the PEG-containing copolymers resulted in a higher percentage of oxygen and hydrogen in the polymer, including decreased crystallinity and molecular weight along with increased water uptake capacity and hydrophilicity. Indeed, the water contact angle and water uptake parameters were significantly higher in PHB-PEG: 24% decrease in contact angle and 34-fold increase in water uptake in comparison with PHB. Moreover, the calculated surface free energy components indicated that the polar component played an important role on surface free energy of PHB-PEG copolymer in contrast with PHB. The polar component of surface free energy was 4-fold higher in PHB-PEG copolymer in comparison with PHB, leading to a maximal value of total surface free energy.

### Surface morphology of copolymers films

**Figure 3:**
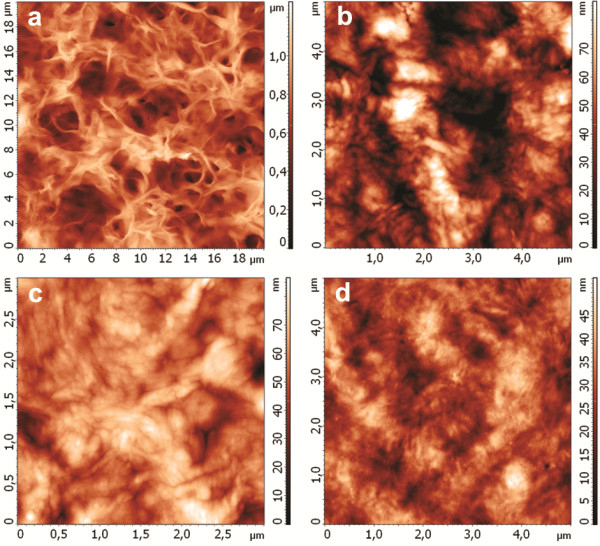
**AFM microphotography of film surface of produced PHAs.** AFM microphotography of film surface of produced PHAs: (**a**) PHB, “rough” surface; (**b**) “smooth” surface; (**c**) PHB-HV, “smooth” surface; (**d**) PHB-PEG, “smooth” surface.

The film casting procedure allowed distinction of morphology between two surfaces when one plane of the polymer was adjacent to the glass plate and the other plane was exposed to air. Part “a” of Figure 3 clearly illustrates that the surface exposed to air has a roughness with plentiful pores characterized by a depth of 500–700 nm. As seen in Part “b” of Figure 3, the opposite side of the film that was in contact with the glass was characterized by minor texture and by shallower pores (as small as 100 nm). At higher magnifications (data not shown) in certain localities, the stacks of polymer crystallites with widths of about 100 nm and lengths between 500–800 nm were visible.

The variance of characteristics was related to solvent desorption conditions during its evaporation from the cast film. During chloroform evaporation from the air-exposed surface, the flux formed additional channels (viz. the pores), which were fixed as far as the film solidified and crystallized. Contrarily, during evaporation the morphology and texture of the opposite side of the film exposed to the glass support were not subjected to the impact of solvent transport. The morphology of the latter surface predominantly depended on energy interaction conditions (interface glass-biopolymer tension) [31].

The surface morphology of various biopolymer films did not differ greatly. As seen in Table 4, the average roughness of “smooth” surface of PHB copolymers films decreased slightly relative to the PHB homopolymer; “rough” surface of polymer films did not differ significantly.

**Table 4:** **Average roughness of polymer film surface (R**_**a**_**)**

**Polymer**	**“Smooth surface”**	**“Rough” surface**
	**(R**_**a**_**, nm ± SD)**	**(R**_**a**_**, nm ± SD)**
PHB	15.0 ± 2.0	130 ± 19
PHB-HV	9.4 ± 1.5^#^	93 ± 21
PHB-PEG	6.6 ± 1.4^#^	114 ± 23

### Protein adsorption

**Figure 4:**
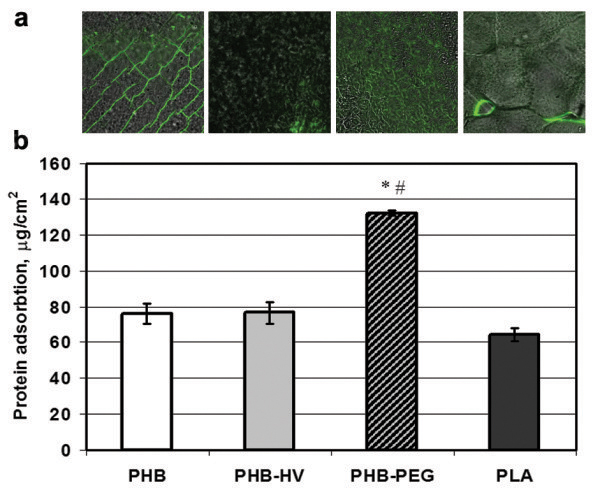
**The protein adsorption on the polymer surface.** The protein adsorption on the polymer surface: (**a**) FITC-BSA distribution on the surface of PHB, PHB-HV, PHB-PEG, and PLA polymer films visualized by florescence microscopy; (**b**) the absorption of proteins from bovine fetal serum on the PLA, PHB, PHB-HV and PHB-PEG polymer films. Data were shown as Mean ± SD (n = 6); * vs PLA, # vs PHB, p < 0.01.

Figure 4 shows the protein adsorption, which was detected on the films of PHB, PHB-HV and PHB-PEG. PHB-PEG films presented the highest protein absorption ability more than 2-fold greater than PLA, PHB and PHB-HV films, as shown in Figure 4b. The highest protein adsorption to PHB-PEG films is probably connected with the maximal water contact angles, the water uptake, the total surface free energy and its polar component of this copolymer. This result is in agreement with the observation made by Collier et al. [43] that albumin adsorbs preferably to hydrophilic surfaces, which seems to correlate with the wettability results of our polymer films (Table 3). A positive correlation between polymer surface hydrophilicity (as well as total surface free energy and its polar component) and protein adsorption to polymer surface was shown also in other studies [34,44]. Moreover, analysis of protein adsorption by fluorescence microscopy demonstrated the difference in morphological distribution of adsorbed FITC-BSA on the polymer surface. As seen in Figure 4a, on the PLA and PHB films surface the protein adsorbed greatly onto defects of polymer surface, while adsorption of FITC-BSA on PHB-HV and PHB-PEG films surface was uniform. The uniform distribution of adsorbed protein on the PHB-HV and PHB-PEG film surface is associated probably with decreased crystallinity of the polymers, while the greater amount of adsorbed protein on the PHB-PEG film compared to PHB-HV film is associated with greater PHB-PEG surface hydrophilicity.

### Biocompatibility of produced biopolymers in vitro

**Figure 5:**
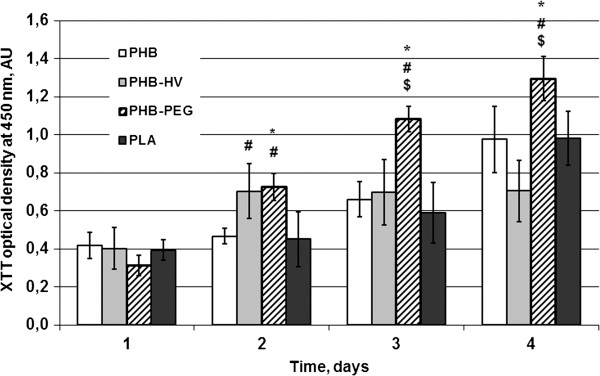
**Adhesion and cell proliferation on biopolymer films.** Adhesion and cell proliferation on tested biopolymer films evaluated by XTT test: PHB, PHB-HV, PHB-PEG, and PLA. Data were shown as Mean ± SD; n = 12 (PHB, PHB-HV), n = 8 (PHB-PEG, PLA); ^*^ vs PLA, ^#^ vs PHB, ^$^ PHB-PEG vs PHB-HV, p < 0.05.

Cell cytotoxicity testing is one of the critical factors affecting the biomedical application of polymers [1,2]. Here, we used COS-7 fibroblasts to demonstrate that the naturally hydrophobic PHB-surface could be modified into a more cell-compatible surface by 3HV or PEG modification of the PHB biopolymer. Cells exhibited remarkable growth and proliferation after only 24 h incubation on different polymers films compared with PLA (control), as measured by the XTT assay. Cell adhesion of cells on PHB films showed a tendency to be stronger than on PLA films at 2–4 days, but this difference was not significant. There was also no significant difference in cell adhesion between the PHB-HV and PLA as well as between the PHB-HV and PHB. However, as indicated in Figure 5, there was a distinct difference between the cell attachment on the PHB homopolymer and PEG-modified PHB copolymer. Figure 5 illustrates that COS-7 fibroblasts attached to tested PHB-PEG films displayed significantly stronger adhesion compared to their interaction with PHB, PHB-HV and PLA films at 2, 3 and 4 days incubation. After 4 days incubation, the highest XTT values were observed with PHB-PEG film, which was almost equal to that of polystyrene plate, whereas cells grown on other tested films showed cell adhesion approximately two to three-fold lower than that of TCPs (data not shown).

As commonly known, protein adsorption plays an important role in cell adhesion. Generally, cells grow on a layer of protein that interacts with cellular receptors and the hydrophilic surface is favorable to adhesion and growth of cells [34,45]. The more hydrophilic surface of PHB-PEG films facilitated absorption of proteins. Indeed, PHB-PEG display more hydrophilic properties (that were evaluated by measurement of water contact angle and water uptake parameters) relative to PHB, PHB-HV and PLA. Moreover, as seen in fluorescent microscopy microphotographs of Figure 4a, irregular protein adsorption on PHB films can hinder protein layer formation as opposed to PHB-PEG film, which is covered with protein uniformly. However, not only surface hydrophilicity but also surface morphology effects cell attachment and proliferation. Different cells prefer different surfaces, e.g., it was found that fibroblasts preferred to attach to a relatively rougher surface, while epithelial cells only attached to the smoothest surface [46-48]. But COS-7 cells grew on the “rough” surface of polymer films, the roughness of this surface of various polymer films did not differ, as shown in Table 4. Indeed, cell adhesion of COS-7 fibroblasts to more hydrophilic (compared with PHB) PEG-modified PHB copolymer was stronger in opposition to cell adhesion to more hydrophobic (compared with PHB) PHB-HV copolymer. This data correlates with biocompatibility of PHB/PHB-HHx blends, which depends on surface hydrophilicity of polymer films [34]. It was shown that PHB/PEG blending improved cell compatibility and decreased blood coagulation and platelet adhesion to biopolymers compared to pure PHB. Improved cell- and hemo-compatibility was also associated with increased hydrophilicity of PHB/PEG blends [20]. Thus, our results demonstrate that the PEG-modified PHB copolymer possesses the ability to maintain cell viability and growth, thereby indicating the non-toxicity of the PEG-modified PHB copolymer to COS-7 fibroblasts.

## Conclusions

Taken together, our results indicate that introduction of carboxylic acids and EG-derivates into A. chroococcum 7B culture represent a viable approach to the production of PHA copolymers. Despite of low EG-monomers content in bacterial origin PHB-PEG copolymer, this polymer demonstrated significant improvement in biocompatibility in contrast to PHB and PHB-HV copolymers, which may be coupled with increased protein adsorption and hydrophilicity of PEG-tailing copolymer. Currently, we are working to adjust the materials by tailoring the compositions achieve a balance between biocompatibility, physicochemical properties, processing ability and device fabrication.

## Abbreviations

PHA: Poly(3-hydroxyalkanoate); PHB: Poly(3-hydroxybutyrate); PHB-HV: Copolymer, poly(3-hydroxybutyrate-co-3-hydroxyvalerate); 3HV: 3-hydroxyvalerate; PEG: Poly(ethylene glycol); PHB-PEG: Copolymer, poly(3-hydroxybutyrate)-poly(ethylene glycol); XTT: (2,3-bis-(2-methoxy-4-nitro-5-sulfophenyl)-2H-tetrazolium-5-carboxanilide) – reagent for Cell Viability Assay; VA: Valeric acid; SA: Sodium acetate; PLA: Polylactic acid; Mw: Molecular weight; AFM: Atomic force microscopy; 1H NMR: Proton nuclear magnetic resonance; DSC: Differential scanning calorimetry.

## Competing interests

Financial competing interests.

In the past five years have you received reimbursements, fees, funding, or salary from an organization that may in any way gain or lose financially from the publication of this manuscript, either now or in the future? Is such an organization financing this manuscript (including the article-processing charge)? If so, please specify.

No.

Do you hold any stocks or shares in an organization that may in any way gain or lose financially from the publication of this manuscript, either now or in the future? If so, please specify.

No.

Do you hold or are you currently applying for any patents relating to the content of the manuscript? Have you received reimbursements, fees, funding, or salary from an organization that holds or has applied for patents relating to the content of the manuscript? If so, please specify.

No.

Do you have any other financial competing interests? If so, please specify.

No.

Non-financial competing interests.

Are there any non-financial competing interests (political, personal, religious, ideological, academic, intellectual, commercial or any other) to declare in relation to this manuscript? If so, please specify.

No.

## Authors’ contributions

BAP1 performed the analysis and interpretation of data, participated in conception and design and drafted the manuscript. YSG carried out the analysis of water related properties of copolymers (contact angle, water uptake) and drafted the manuscript. ZII carried out the COS-7 fibroblast cultivating and growth on polymers and XTT-based cell viability assay. BAP2 performed the polymer films preparation and study of the polymer composition by ^1^H nuclear magnetic resonance. BDV performed the calculation, interpretation and processing data on atomic force microscopy. MVL carried out A. chroococcum strain 7B cultivating and PHB-PEG biosynthesis. MTK carried out extraction and purification of polymers and molecular weight determination. KEP performed the analysis of physico-thermal properties of copolymers by differential scanning calorimetry. SOV carried out the fluorescence microscopy of protein adsorption by operational work on the microscope. FAV performed fluorescence microscopy of protein adsorption by interpretation and processing data and interpreted the data on cell proliferation and protein absorption on the copolymers. VVV participated in calculations, statistical analysis, illustrations and tables design. ZAL carried out protein adsorption analysis by quantitative method. EYM carried out the atomic force microscopy by operational work on the microscope. BGA participated in conception and design, drafted the section «Growth conditions», performed A. chroococcum strain 7B cultivating and PHB and PHB-HV biosynthesis. SKV participated in conception and design and revised it critically for important intellectual content. KMP have given final approval of the version to be published. All authors read and approved the final manuscript.
